# Supplementary material for: Differential Effects of Extracellular Vesicles from Two Different Glioblastomas on Normal Human Brain Cells
Source: Neurol Int. 2024 Nov 6;16(6):1355–84. doi: 10.3390/neurolint16060103 (PMC11587087; doi:10.3390/neurolint16060103)
Supplement: Supplementary file 1 [file neurolint-16-00103-s001.zip › neurolint-3220349-supplementary-proof.pdf]

neurology international

MDPI

Supplemental Materials

Supplementary Material: Differential Effects of Extracellular Vesicles from Two Different Glioblastomas on Normal Human Brain Cells

MAIN TEXT

This Supplementary section contains Supplementary Figures 1-10. Supplementary Tables 1-4 are supplied separately as Excel or Word files for better accessibility.

Supplemental Table S1:.Mass Spectrometry-Based Proteomic Analyses of F3-8 or G17-1 GBM EVs. MS details are found in the Materials and Methods section of the main text.

Supplemental Table S2: Transcriptome: Astrocytes treated with F3-8 GBM EVs.

Total RNA was extracted from astrocytes using RNeasy Plus Micro Kits (74034; QIAGEN, Germantown, MD, USA). RNA quality/quantity was checked with a 2100 Bioanalyzer (Agilent Technologies, Santa Clara, CA, USA). RNA Integrity Numbers were 9.9 or higher. Samples were then handled by the University of Colorado Anschutz Genomics and Microarray Core for microarray analysis using a Human Clariom D chip (902922; Applied Biosystems via ThermoFisher). This array has over 540,000 transcripts on it including mRNAs, miRNAs, lncRNAs, and splice variants. RNAs were analyzed using Transcriptome Analysis Console (TAC) 4.0.1 (ThermoFisher).

Supplementary Table S3: Transcriptome: Astrocytes treated with G17-1 GBM EVs.

Total RNA was extracted from astrocytes using RNeasy Plus Micro Kits (74034; QIAGEN, Germantown, MD, USA). RNA quality/quantity was checked with a 2100 Bioanalyzer (Agilent Technologies, Santa Clara, CA, USA). RNA Integrity Numbers were 9.9 or higher. Samples were then handled by the University of Colorado Anschutz Genomics and Microarray Core for microarray analysis using a Human Clariom D chip (902922; Applied Biosystems via ThermoFisher). This array has over 540,000 transcripts on it including mRNAs, miRNAs, lncRNAs, and splice variants. RNAs were analyzed using Transcriptome Analysis Console (TAC) 4.0.1 (ThermoFisher).

Supplementary Table S4: Mass Spectrometry-Based Proteomic Analyses of Astrocytes treated with F3-8 or G17-1 GBM EVs. MS details are found in the Materials and Methods section of the main text.

Supplementary Table S5: Phosphorylation Sites on Proteins in Creative Biolabs Human Phospho Kinase Antibody Array AbAr-0225-Y

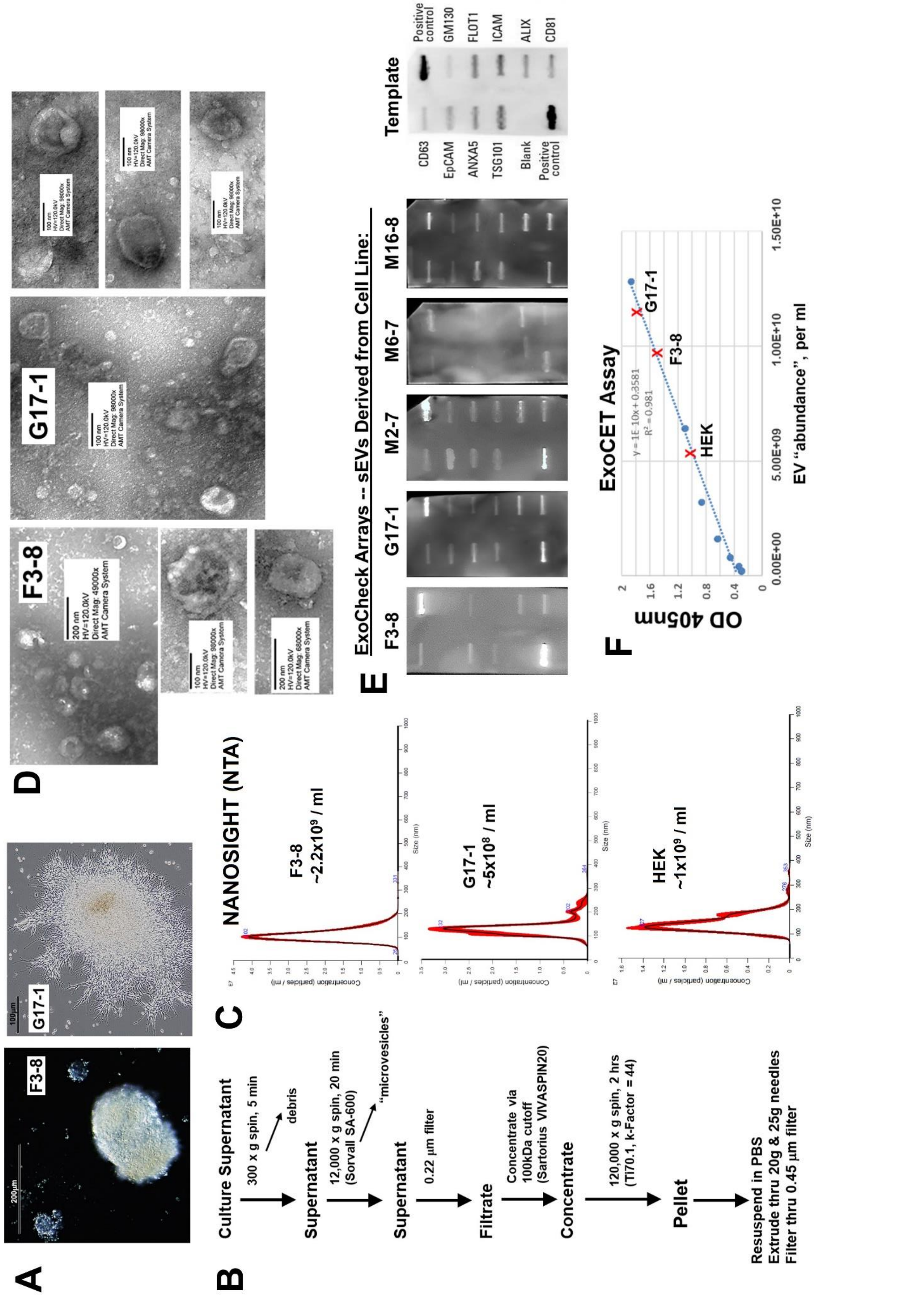
A
F3-8
200µm
G17-1
100µm
B
Culture Supernatant
300 x g spin, 5 min
debris
Supernatant
12,000 x g spin, 20 min
(Sorvall SA-600)
"microvesicles"
Supernatant
0.22 µm filter
Filtrate
Concentrate via
100kDa cutoff
(Sartorius VIVASPIN20)
Concentrate
120,000 x g spin, 2 hrs
(Ti70.1, k-Factor = 44)
Pellet
Resuspend in PBS
Extrude thru 20g & 25g needles
Filter thru 0.45 µm filter
C
NANOSIGHT (NTA)
F3-8
~2.2x10⁹ / ml
G17-1
~5x10⁸ / ml
HEK
~1x10⁹ / ml
Concentration (particles / ml)
Size (nm)
D
F3-8
G17-1
E
ExoCheck Arrays -- sEVs Derived from Cell Line:
F3-8
G17-1
M2-7
M6-7
M16-8
Template
CD63
EpCAM
ANXA5
TSG101
Blank
Positive control
Positive control
GM130
FLOT1
ICAM
ALIX
CD81
F
ExoCET Assay
OD 405nm
EV "abundance", per ml
y = 1E-10x + 0.3581
R² = 0.981
G17-1
F3-8
HEK

Supplemental Figure S1: GBM cell lines used in the study, and extracellular vesicle production and characterization. **A**: F3-8 (left) and G17-1 (right) spheroids grown under stem cell-like conditions. Scale bars = 200µm for F3-8 image, 100µm for G17-1 image. **B**: Schematic of steps involved in EV isolation. **C**: Nanosight nanoparticle tracking analysis (NTA) of F3-8 EVs (Stats: Merged Data; Mean:110.6 nm, Mode: 101.3 nm, SD: 25.5 nm), of G17-1 EVs (Stats: Merged Data; Mean: 135.8 nm, Mode: 131.3 nm, SD: 34.9 nm), and of HEK293 EVs (Stats: Merged Data; Mean: 144.4 nm, Mode: 126.9 nm, SD: 33.9 nm) with concentrations listed. **D**: Transmission electron microscopy of F3-8 EVs (left, top, and two beneath) and G17-1 EVs (right panels) with scale bars/magnification listed on each image. **E**: ExoCheck arrays showing putative typical EV markers for lysed EVs from cell lines: F3-8; G17-1; M2-7; M6-7; M16-8. The template is on the far right. **F**: ExoCET (acetylcholinesterase) assay showing enzymatic activity and putative quantification for HEK, F3-8, and G17-1 EVs.

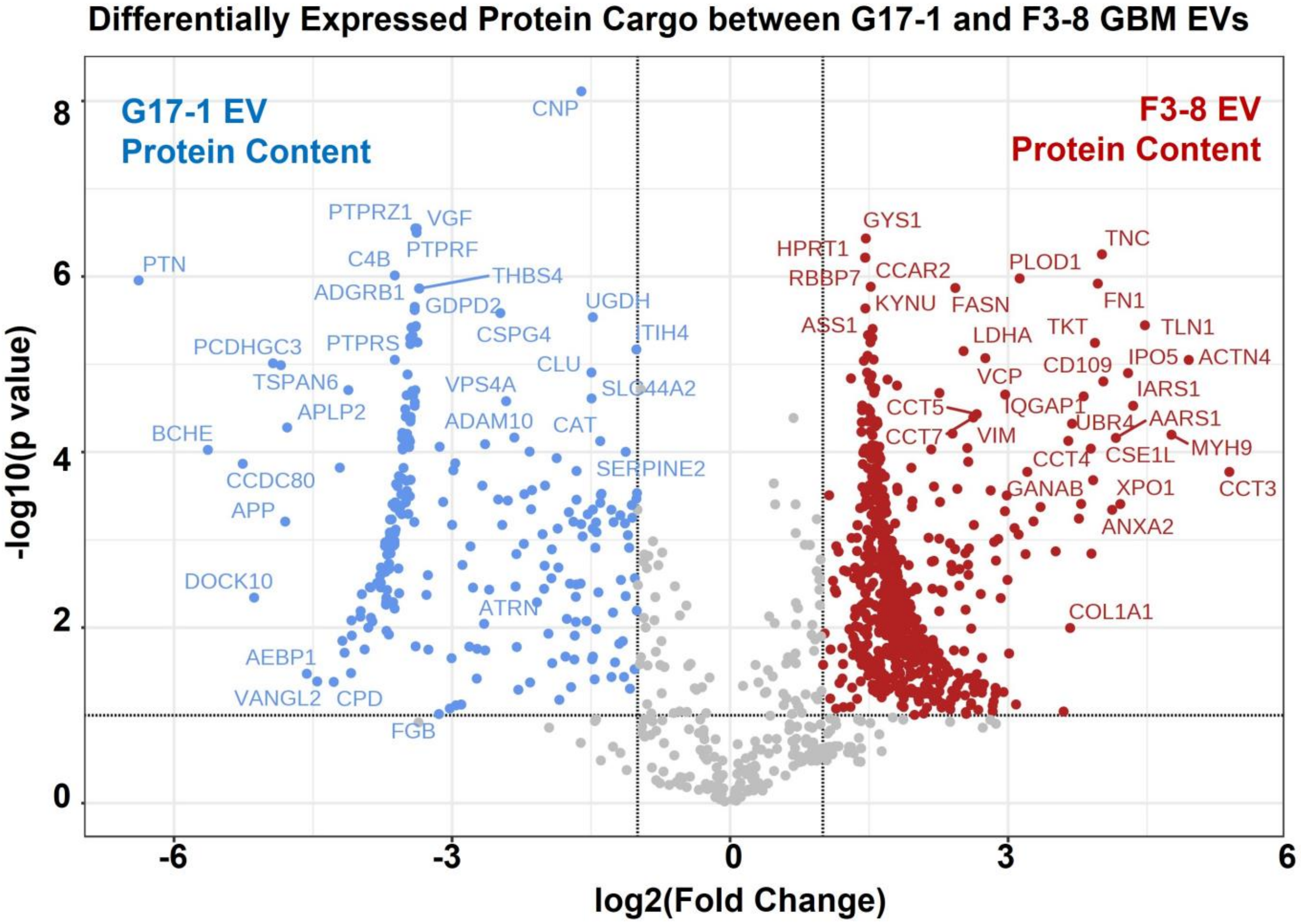

Supplemental Figure S2: Volcano plot showing statistically significant differences in protein expression in (left, in blue) G17-1 GBM EVs, and (right, in red) F3-8 GBM EVs. Data are display based on Metaboanalyst 5.0

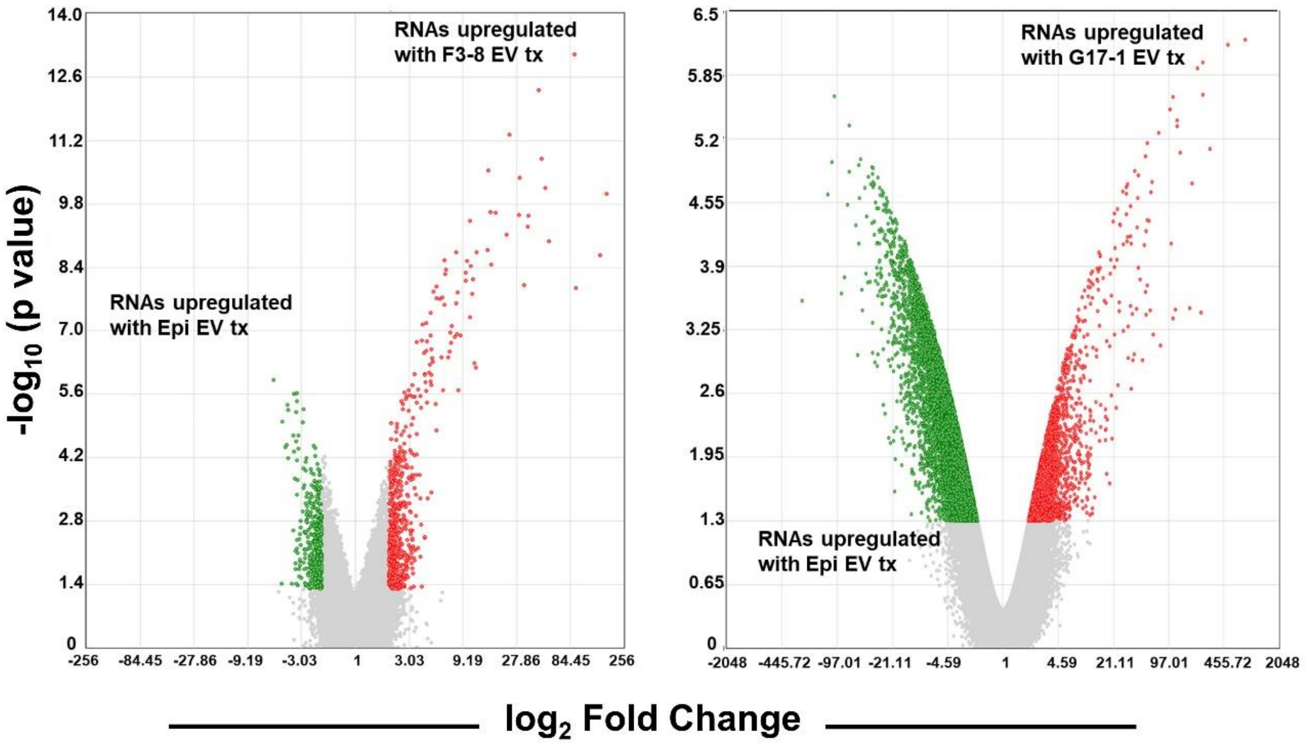

Supplemental Figure S3: Transcriptomic data volcano graphs for astrocytes treated with GBM EVs, Astrocytes were treated with GBM F3-8 EVs (left panel) or G17-1 EVs (right panel). Control treatment ("tx") was with human epithelial (Epi) cell EVs. Astrocytes were extracted and the transcriptomes were analyzed on Human Clariom D chips; volcano graphs were generated in Transcriptome Analysis Console (TAC) 4.0.1 (ThermoFisher). Red dots show RNAs significantly upregulated with GBM EV treatments, while green dots show RNAs significantly upregulated with epithelial EV treatments.

IPA Modified Transcriptomic Summary, Astrocytes Treated with F3-8 EVs

Top Canonical Pathways

Name	p-value	Overlap
Interferon Signaling	3.42E-16	38.9 % 14/36
Antigen Presentation Pathway	8.44E-16	36.8 % 14/38
Th1 Pathway	1.02E-12	14.4 % 19/132
Th1 and Th2 Activation Pathway	5.18E-12	11.5 % 21/182
OX40 Signaling Pathway	5.90E-12	23.6 % 13/55

Top Diseases and Bio Functions

Diseases and Disorders

Name	p-value range	# Molecules
Antimicrobial Response	3.87E-11 - 1.11E-35	42
Inflammatory Response	3.62E-05 - 1.11E-35	153
Immunological Disease	2.77E-05 - 2.39E-28	157
Dermatological Diseases and Conditions	3.35E-05 - 1.65E-26	94
Organismal Injury and Abnormalities	3.58E-05 - 1.65E-26	314

Molecular and Cellular Functions

Name	p-value range	# Molecules
Cellular Movement	3.62E-05 - 5.49E-17	109
Cell Death and Survival	3.62E-05 - 1.08E-16	127
Cellular Development	3.43E-05 - 1.59E-15	118
Cellular Growth and Proliferation	3.43E-05 - 1.59E-15	120
Cell-To-Cell Signaling and Interaction	3.62E-05 - 4.58E-14	106

Physiological System Development and Function

Name	p-value range	# Molecules
Lymphoid Tissue Structure and Development	3.43E-05 - 1.62E-15	57
Hematological System Development and Function	3.62E-05 - 2.18E-14	78
Immune Cell Trafficking	3.62E-05 - 3.06E-14	62
Cell-mediated Immune Response	2.90E-05 - 4.46E-12	36
Hematopoiesis	2.07E-05 - 8.37E-12	36

Top Regulator Effect Networks

ID	Regulators	Disease & Functions	Consistency Score
1	poly rI:rC-RNA	Immune response of cells	4.146
2	IL17A	Inflammatory response	3.873
3	IL1B	Chemotaxis of granulocytes	3.618
4	IL1B	Chemotaxis of myeloid cells	3.618
5	IL1A	Invasion of cells	3.615

Top Networks

ID	Associated Network Functions	Score
1	Cell Cycle, Cell-To-Cell Signaling and Interaction, Cellular Development	41
2	Cell Signaling, Post-Translational Modification, Protein Folding	41
3	Antimicrobial Response, Inflammatory Response, Infectious Diseases	38
4	Infectious Diseases, Cell Morphology, Hematological System Development and Function	32
5	Endocrine System Disorders, Gastrointestinal Disease, Immunological Disease	26

Supplemental Figure S4: Ingenuity Pathway Analysis (IPA) modified summary reduced to 1 page, IPA modified summary for transcriptomics of astrocytes treated with GBM F3-8 EVs.

IPA Modified Transcriptomic Summary, Astrocytes Treated with G17-1 EVs

Top Canonical Pathways

Name	p-value	Overlap
Hepatic Fibrosis / Hepatic Stellate Cell Activation	2.83E-07	21.4 % 39/182
UDP-N-acetyl-D-glucosamine Biosynthesis II	3.33E-05	83.3 % 5/6
Synaptogenesis Signaling Pathway	3.44E-05	16.2 % 50/308
Osteoarthritis Pathway	4.48E-05	17.9 % 37/207
Cellular Effects of Sildenafil (Viagra)	5.14E-05	20.6 % 26/126

Top Diseases and Bio Functions

Diseases and Disorders

Name	p-value range	# Molecules
Cancer	1.12E-05 - 8.45E-28	1699
Organismal Injury and Abnormalities	1.12E-05 - 8.45E-28	1723
Gastrointestinal Disease	1.12E-05 - 3.60E-27	1527
Reproductive System Disease	3.70E-06 - 5.40E-26	1159
Neurological Disease	8.17E-06 - 1.48E-23	542

Molecular and Cellular Functions

Name	p-value range	# Molecules
Cellular Movement	1.03E-05 - 1.31E-25	450
Cell Death and Survival	1.08E-05 - 7.27E-24	545
Cell-To-Cell Signaling and Interaction	1.14E-05 - 3.13E-13	373
Cellular Assembly and Organization	9.11E-06 - 3.13E-13	254
Cell Cycle	4.45E-06 - 4.38E-13	196

Physiological System Development and Function

Name	p-value range	# Molecules
Cardiovascular System Development and Function	1.07E-05 - 2.62E-14	169
Organismal Development	1.07E-05 - 5.77E-14	150
Hematological System Development and Function	1.03E-05 - 7.14E-14	179
Immune Cell Trafficking	1.03E-05 - 7.14E-14	153
Tissue Development	1.07E-05 - 2.04E-12	259

Top Regulator Effect Networks

ID	Regulators	Disease & Functions	Consistency Score
1	ACTN4,Ap1,CAMP,CCL2,COL18A1,Collagen type II,F2RL1 (+22 more)	Activation of cells,Allergy,Attraction of cells (+5 more)	32.065
2	Ap1,CAMP,CD14,cytokine,ERK,Fibrinogen,HMGB1,IKBKB (+7 more)	Attraction of cells,Liver Damage,Psoriasis (+2 more)	30.486
3	Ap1,CAMP,ERK,HMGB1,IKBKB,IL1,IL17A,IL17F,IL17R (+7 more)	Allergy,Attraction of cells,Psoriasis (+2 more)	23.308
4	BSG,CAMP,Collagen type II,ERK1/2,F2R,IKBKB,IL17R (+2 more)	Allergy,Liver Damage,Psoriasis (+2 more)	12.81
5	BSG,CAMP,Collagen type II,ERK1/2,F2R,IKBKB,IL17R (+3 more)	Allergy,Attraction of cells,Liver Damage,Psoriasis (+2 more)	12.413

Top Networks

ID	Associated Network Functions	Score
1	Cellular Assembly and Organization, DNA Replication, Recombination, and Repair, Cell Cycle	37
2	Cell Death and Survival, Cancer, Gastrointestinal Disease	37
3	Cell-To-Cell Signaling and Interaction, Nervous System Development and Function, Cell Cycle	35
4	Cell-To-Cell Signaling and Interaction, Cancer, Organismal Injury and Abnormalities	35
5	Cell-To-Cell Signaling and Interaction, Cellular Assembly and Organization, Nervous System Development and Function	32

Supplemental Figure S5: IPA modified summary reduced to 1 page, IPA modified summary for transcriptomics of astrocytes treated with GBM G17-1 EVs.

Ingenuity Pathway Analysis Modified Proteomic Summary, Astrocytes Treated with F3-8 EVs

Top Canonical Pathways

Name	p-value	Overlap
Role of Hypercytokinemia/hyperchemokinemia in the Pathogenesis of Influenza	3.97E-10	14.9 % 13/87
Hepatic Fibrosis Signaling Pathway	1.27E-09	6.0 % 26/433
Interferon Signaling	1.72E-09	25.0 % 9/36
Caveolar-mediated Endocytosis Signaling	1.46E-08	14.3 % 11/77
Sertoli Cell-Sertoli Cell Junction Signaling	1.07E-07	7.5 % 16/214

Top Diseases and Bio Functions

Diseases and Disorders

Name	p-value range	# Molecules
Cancer	3.46E-06 - 3.34E-34	363
Organismal Injury and Abnormalities	3.51E-06 - 3.34E-34	369
Endocrine System Disorders	2.65E-06 - 1.11E-24	325
Gastrointestinal Disease	2.90E-06 - 1.22E-23	332
Reproductive System Disease	2.65E-06 - 1.70E-23	293

Molecular and Cellular Functions

Name	p-value range	# Molecules
Cellular Assembly and Organization	2.91E-06 - 2.47E-16	112
Cellular Function and Maintenance	3.48E-06 - 2.47E-16	107
Cell Morphology	2.91E-06 - 2.06E-15	92
Cellular Movement	3.03E-06 - 2.06E-15	117
Cell Death and Survival	3.51E-06 - 2.31E-13	161

Physiological System Development and Function

Name	p-value range	# Molecules
Organismal Survival	1.46E-09 - 5.58E-12	134
Cardiovascular System Development and Function	1.79E-06 - 6.43E-11	94
Connective Tissue Development and Function	1.01E-06 - 2.78E-09	68
Tissue Development	3.53E-06 - 2.78E-09	127
Embryonic Development	9.14E-08 - 6.78E-09	72

Top Regulator Effect Networks

ID	Regulators	Disease & Functions	Consistency Score
1	ACKR2,AIRE,AREG,ATG5,C10orf99,CD276,E2F3,ETV7,FADD (+38 more)	Antiviral response (+5 more)	52.231
2	CD40LG,EBI3,FADD,IFN type 1,Ifnar,IFNAR2,IFNB1 (+22 more)	Antiviral response (+2 more)	37.717
3	ACKR2,BTK,CD276,CGAS,CHUK,CITED2,CLPP,DDX58,DOCK8 (+83 more)	Antiviral response (+3 more)	35.025
4	ALDH2,CCR2,CR1L,F2R,FERMT2,HNF4G (+11 more)	Cell movement of melanoma cell lines (+1 more)	19.667
5	ATM,BCL2L1,CIITA,IPMK,LLGL2,NSUN6,PRKG1,ROR1,SMAD2 (+2 more)	Cell movement of melanoma cell lines (+1 more)	16.148

Top Networks

ID	Associated Network Functions	Score
1	Connective Tissue Disorders, Developmental Disorder, Gastrointestinal Disease	57
2	Developmental Disorder, Hereditary Disorder, Metabolic Disease	52
3	Cancer, Gastrointestinal Disease, Hepatic System Disease	52
4	Infectious Diseases, Organismal Injury and Abnormalities, Cardiovascular Disease	44
5	Cell Signaling, Post-Translational Modification, Protein Synthesis	33

Supplemental Figure S6: IPA modified summary reduced to 1 page, IPA modified summary for proteomics of astrocytes treated with GBM F3-8 EVs.

Ingenuity Pathway Analysis Modified Proteomic Summary, Astrocytes Treated with G17-1 EVs

Top Canonical Pathways

Name	p-value	Overlap
Remodeling of Epithelial Adherens Junctions	9.40E-09	16.2 % 11/68
Coronavirus Replication Pathway	4.79E-07	17.8 % 8/45
Sertoli Cell-Sertoli Cell Junction Signaling	1.14E-06	7.3 % 15/206
Germ Cell-Sertoli Cell Junction Signaling	3.44E-06	7.6 % 13/170
ILK Signaling	2.10E-05	6.5 % 13/201

Top Diseases and Bio Functions

Diseases and Disorders

Name	p-value range	# Molecules
Cancer	9.45E-06 - 9.25E-24	364
Organismal Injury and Abnormalities	9.45E-06 - 9.25E-24	368
Endocrine System Disorders	9.45E-06 - 3.93E-19	319
Gastrointestinal Disease	9.45E-06 - 1.07E-17	328
Reproductive System Disease	9.45E-06 - 2.26E-12	266

Molecular and Cellular Functions

Name	p-value range	# Molecules
Cellular Assembly and Organization	7.46E-06 - 9.42E-12	112
Cellular Function and Maintenance	9.00E-07 - 9.42E-12	92
Cell Morphology	9.00E-07 - 3.94E-09	67
Cellular Movement	9.08E-06 - 3.94E-09	81
Cellular Compromise	6.60E-09 - 6.60E-09	6

Physiological System Development and Function

Name	p-value range	# Molecules
Organismal Survival	1.19E-06 - 2.91E-08	125
Nervous System Development and Function	1.17E-06 - 2.19E-07	33
Tissue Development	7.46E-06 - 2.19E-07	58
Embryonic Development	8.21E-06 - 8.21E-06	57
Organismal Development	8.21E-06 - 8.21E-06	57

Top Regulator Effect Networks

ID	Regulators	Disease & Functions	Consistency Score
1	CAV1,CX3CR1,EDN1,ERBB3,F2R,FGF2,HMG20A,IPMK (+9 more)	Anogenital cancer,Cell transformation (+5 more)	11.143
2	ADAM17,CCR2 (+2 more)	Cell transformation (+4 more)	7.542
3	CCN1,SORL1,SPDEF	Cell transformation (+2 more)	7.236
4	HNF4G,let-7a-5p (and other miRNAs w/seed GAGGUAG) (+5 more)	Cell spreading,Migration of tumor cell lines	5.421
5	FGF8,miR-34a-5p (and other miRNAs w/seed GGCAGUG)	Metastasis	1.633

Top Networks

ID	Associated Network Functions	Score
1	Hereditary Disorder, Metabolic Disease, Organismal Injury and Abnormalities	57
2	Cancer, Gastrointestinal Disease, Organismal Injury and Abnormalities	57
3	Cellular Compromise, Embryonic Development, Organ Development	49
4	RNA Post-Transcriptional Modification, Connective Tissue Disorders, Developmental Disorder	46
5	Cancer, Hematological Disease, Immunological Disease	46

Supplemental Figure S7: IPA modified summary reduced to 1 page, IPA modified summary for proteomics of astrocytes treated with GBM G17-1 EVs.

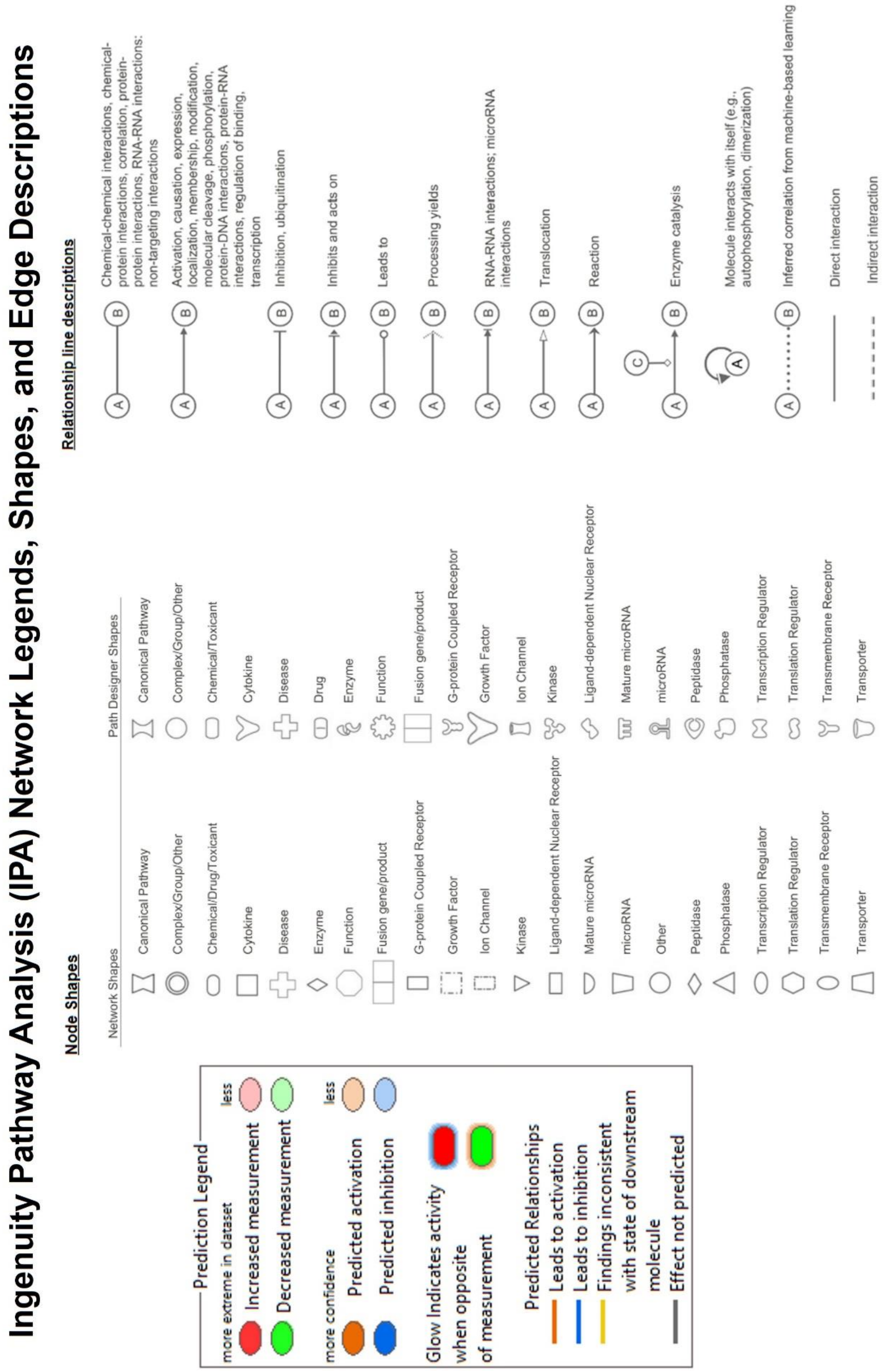

Supplemental Figure S8: Ingenuity Pathway Analysis (IPA) network legends, shapes, and edge descriptions.

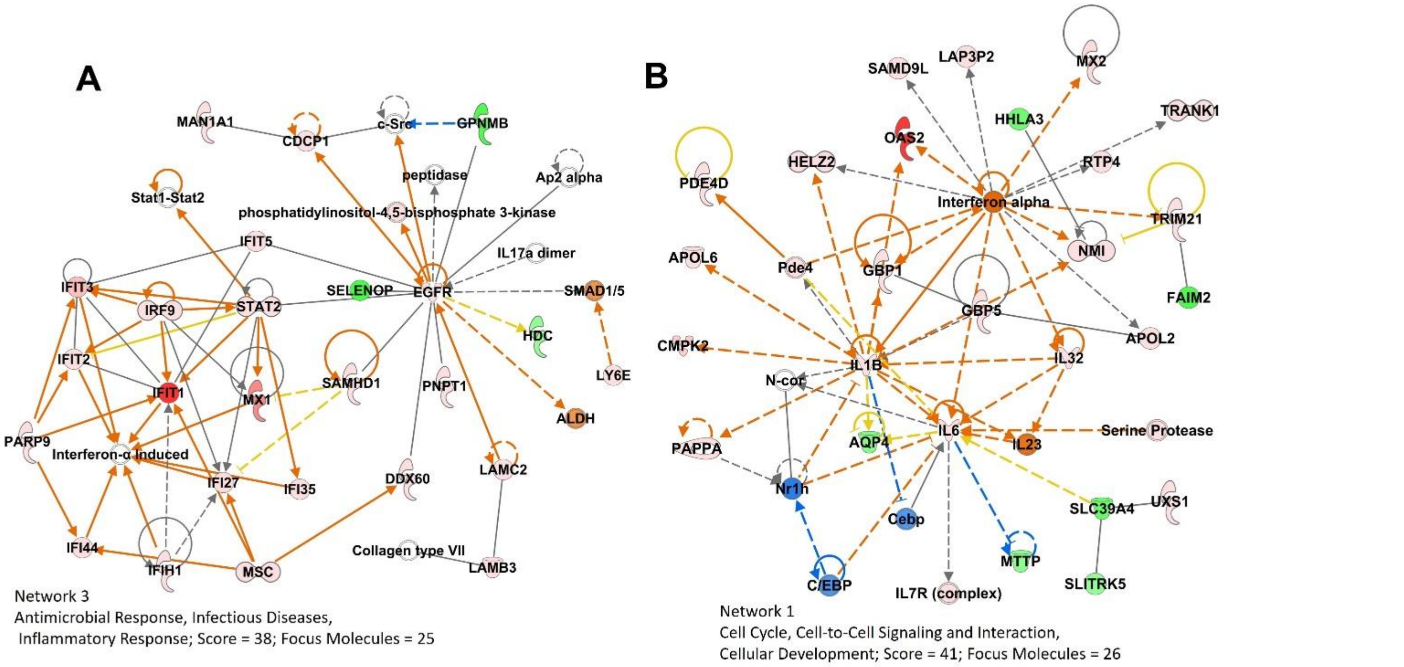

Supplemental Figure S9: Relevant IPA-derived networks from transcriptomics, astrocytes treated with GBM F3-8 EVs. **A**: Network 3: "Antimicrobial Response, Infectious Diseases, Inflammatory Response"; Score = 38; Focus Molecules = 25. **B**: Network 1: "Cell Cycle, Cell-to-Cell Signaling and Interaction, Cellular Development"; Score = 41; Focus Molecules = 26. Score and Focus Molecule definitions are the same as in Figures 2, 3.

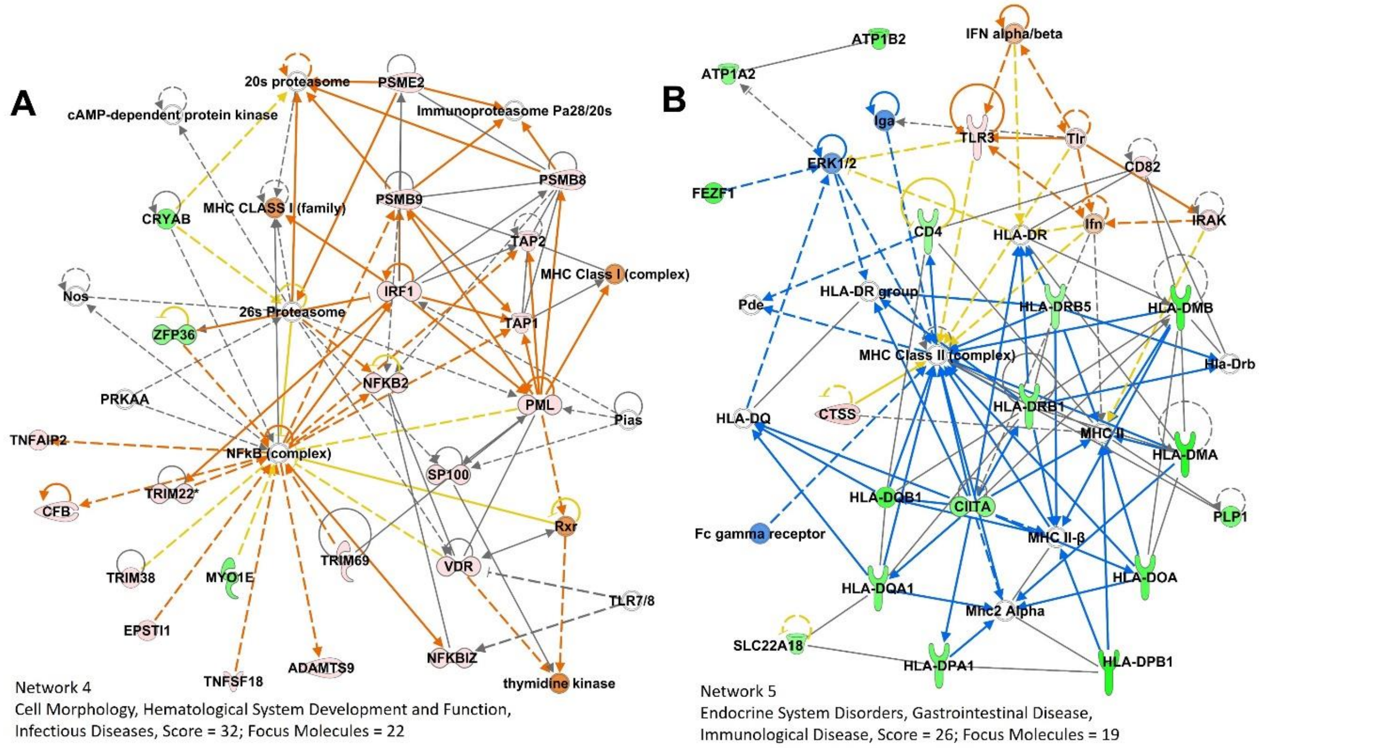

Supplemental Figure S10: Additional relevant IPA-derived networks from transcriptomics, astrocytes treated with GBM F3-8 EVs. **A**: Network 4: "Cell Morphology, Hematological System Development and Function, Infectious Diseases"; Score = 32; Focus Molecules = 22. **B**: Network 5: "Endocrine System Disorders, Gastrointestinal Disease, Immunological Disease"; Score = 26; Focus Molecules = 19. Score and Focus Molecule definitions are the same as in Figure 2, 3.

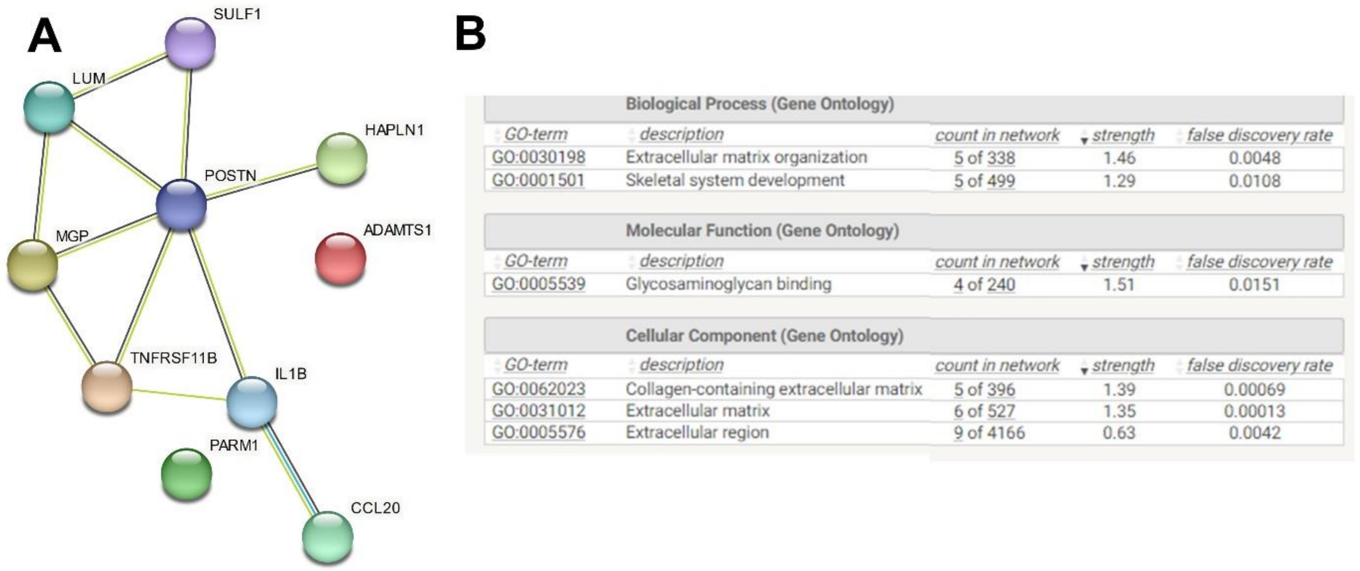

Biological Process (Gene Ontology)

GO-term	description	count in network	strength	false discovery rate
GO:0030198	Extracellular matrix organization	5 of 338	1.46	0.0048
GO:0001501	Skeletal system development	5 of 499	1.29	0.0108

Molecular Function (Gene Ontology)

GO-term	description	count in network	strength	false discovery rate
GO:0005539	Glycosaminoglycan binding	4 of 240	1.51	0.0151

Cellular Component (Gene Ontology)

GO-term	description	count in network	strength	false discovery rate
GO:0062023	Collagen-containing extracellular matrix	5 of 396	1.39	0.00069
GO:0031012	Extracellular matrix	6 of 527	1.35	0.00013
GO:0005576	Extracellular region	9 of 4166	0.63	0.0042

Supplemental Figure 11: STRING analysis of the top 10 upregulated mRNAs from transcriptome of astrocytes treated with GBM G17-1 EVs. **A**: STRING functional interaction protein network of the top 10 most highly upregulated mRNAs (compared to epithelial cell EV-treated astrocytes) from astrocytes treated with G17-1 EVs. **B**: Gene Ontology terms relevant to the mRNAs in (A).

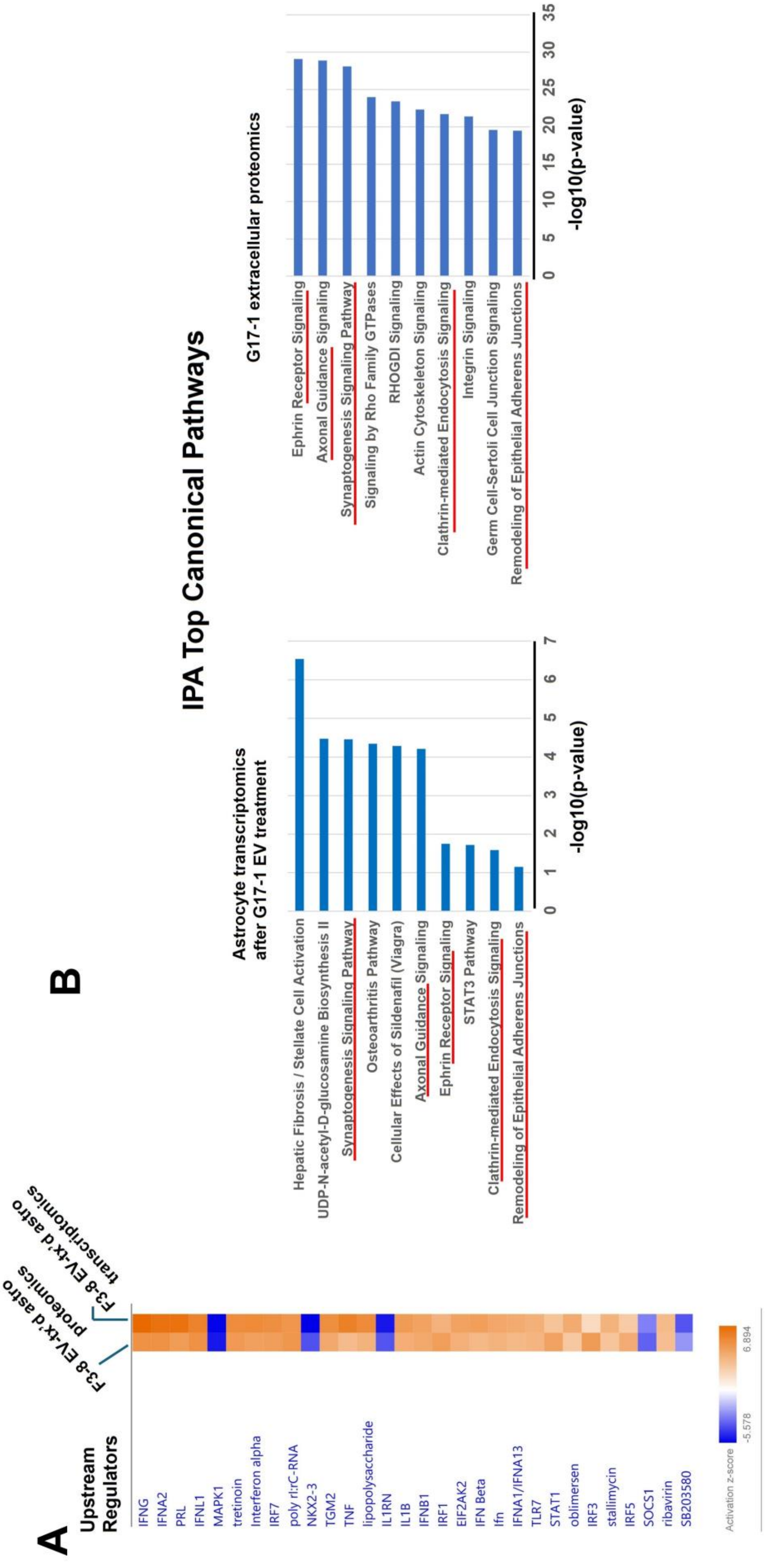
A
Upstream Regulators
F3-8 EV-tx'd astro proteomics
F3-8 EV-tx'd astro transcriptomics
IFNG
IFNA2
PRL
IFNL1
MAPK1
tretinoin
Interferon alpha
IRF7
poly rI:rC-RNA
NKX2-3
TGM2
TNF
lipopolysaccharide
IL1RN
IL1B
IFNB1
IRF1
EIF2AK2
IFN Beta
Ifn
IFNA1/IFNA13
TLR7
STAT1
oblimersen
IRF3
stallimycin
IRF5
SOCS1
ribavirin
SB203580
Activation z-score
-5.578
6.894
B
IPA Top Canonical Pathways
Astrocyte transcriptomics after G17-1 EV treatment
Hepatic Fibrosis / Stellate Cell Activation
UDP-N-acetyl-D-glucosamine Biosynthesis II
Synaptogenesis Signaling Pathway
Osteoarthritis Pathway
Cellular Effects of Sildenafil (Viagra)
Axonal Guidance Signaling
Ephrin Receptor Signaling
STAT3 Pathway
Clathrin-mediated Endocytosis Signaling
Remodeling of Epithelial Adherens Junctions
0 1 2 3 4 5 6 7
-log10(p-value)
G17-1 extracellular proteomics
Ephrin Receptor Signaling
Axonal Guidance Signaling
Synaptogenesis Signaling Pathway
Signaling by Rho Family GTPases
RHOGDI Signaling
Actin Cytoskeleton Signaling
Clathrin-mediated Endocytosis Signaling
Integrin Signaling
Germ Cell-Sertoli Cell Junction Signaling
Remodeling of Epithelial Adherens Junctions
0 5 10 15 20 25 30 35
-log10(p-value)

Supplemental Figure S12: (**A**) IPA Comparison Analysis, Top 30 Upstream Regulators compared between F3-8 EV-treated ("tx'd") astrocytes, proteomic readouts, vs F3-8 EV-treated ("tx'd") astrocytes, transcriptomic readouts. (**B**) Comparison of some top canonical pathways determined by IPA between the transcriptomics of G17-1 EV-treated astrocytes (left graph) and the proteomics of the G17-1 EV treated astrocytes (right graph). Red underline depicts matching canonical pathways between the two.
