# Supplementary material for: Differential Effects of Extracellular Vesicles from Two Different Glioblastomas on Normal Human Brain Cells
Source: Neurol Int. 2024 Nov 6;16(6):1355–84. doi: 10.3390/neurolint16060103 (PMC11587087; doi:10.3390/neurolint16060103)
Supplement: Supplementary file 1 [file neurolint-16-00103-s001.zip › Supp Table S4 phospho sites.pdf]

Supplemental Table 4

Phosphorylation Sites on Proteins in Creative Biolabs Human Phospho-Kinase Antibody Array AbAr-0225-YC

Akt 1/2/3 (S473)
Hck (Y411)
PLC gamma-1 (Y783)
Akt 1/2/3 (T308)
HSP27 (S78/S82)
PRAS40 (T246)
AMPK alpha1 (T183)
HSP60, Pyk2 (Y402)
AMPK alpha2 (T172)
JNK 1/2/3 (T183/Y185, T221/Y223)
RSK1/2/3 (S380)
beta-Catenin, Lck (Y394)
Src (Y419)
Chk-2 (T68)
Lyn (Y397)
STAT2 (Y689)
c-Jun (S63)
MSK1/2 (S376/S360)
STAT3 (S727)
CREB (S133)
p27 (T198)
STAT3 (Y705)
EGFR (Y1086)
p38 alpha (T180/Y182)
STAT5a (Y699)
eNOS (S1177)
p53 (S15)
STAT5a/b (Y699)
ERK1/2 (T202/Y204, T185/Y187)
p53 (S392)
STAT5b (Y699)
FAK (Y397)
p53 (S46)
STAT6 (Y641)
Fgr (Y412)
P70 S6 Kinase (T389)
TOR (S2448)
Fyn (Y420),
p70 S6 Kinase (T421/S424)
WNK-1 (T60)
GSK-3 alpha/beta (S21/S9)
PDGF R beta (Y751)
Yes (Y426)
